# 

**Published:** 1869-08-15

**Authors:** 


### *CONTENTS.*

TRANSLATIONS.

Anatomical and Physiological Observations made upon Decapitated Subjects. By M. Ch. Robin .......... 461
The Intimate Structure of the Axis-Cylinder and of Nerve-Cells .......... 493

COMMUNICATIONS.

The Medical Profession and the Public .......... 476

FOREIGN ITEMS.

Puerperal Hæmorrhage Arrested by Iodine Injections .......... 482
Ovariotomy .......... 482
Bromide of Potassium .......... 483
Traumatic Tetanus .......... 483
Atropine Intoxication .......... 485

EDITORIAL.

Criminal Carelessness .......... 487
Amende Honorable .......... 489
Names Again .......... 489
Bowels .......... 490
Carbolic Acid .......... 491
Discreditable .......... 491
Professional Perspicuity .......... 513
Earth Closets .......... 514
Craig Microscope .......... 515
Fluid Extracts .......... 515

OBITUARY .......... 492

SOCIETY REPORTS.

Chicago Medical Society .......... 506

HOSPITAL REPORTS.

County Hospital Reports .......... 509
Cook County Hospital .......... 512

BOOKS RECEIVED .......... 516

PRICE CURRENT .......... 519

